# Weight perception and mental health disorders among adolescents of central-eastern Tunisia: A cross-sectional study

**DOI:** 10.1371/journal.pone.0308384

**Published:** 2024-10-07

**Authors:** Rim Ghammem, Hela Ghali, Laura Pavicic, Sihem Ben Fredj, Nawel Zammit, Amira Dalhoumi, Rania Bannour, Jihene Maatoug, Hassen Ghannem

**Affiliations:** 1 Department of Epidemiology, “LR19SP03”, Faculty of Medicine of Sousse, Farhat Hached University Hospital, University of Sousse, Sousse, Tunisia; 2 Department of Prevention and Security of Care, Faculty of Medicine of Sousse, Sahloul University Hospital, University of Sousse, Sousse, Tunisia; 3 Master’s Program at Ecole des Hautes Études en Santé Publique (EHESP), Paris, France; 4 Family Medicine, Faculty of Medicine of Sousse, Sousse, Tunisia; Universitas Padjadjaran, INDONESIA

## Abstract

**Background:**

The physical changes that accompany the onset of puberty demand a constant restructuring of the adolescent’s perception of their body and may influence adolescents’ mental health.

**Aim:**

To describe weight status perception and its association with socio-demographic characteristics (SDC) and mental health disorders among adolescents in a low and middle-income country (LMIC).

**Methods:**

We conducted a cross-sectional study in high schools in the urban area of the governorate of Sousse, Tunisia in 2018. We included in our study all students studying in selected classes in selected public high schools and who are consented to participate. Multinomial logistic regression was used to assess the associated factors to weight perception categories.

**Results:**

The total number of students participating was 1399 with a response rate of 86.68%. The female sex was predominant (60.5% versus 39.5%). The mean age was of 17.03 ± 1.51 years. According to multivariate analysis, perceived weight categories were associated with sociodemographic factors such as gender and maternal educational level. Adolescents perceiving themselves as obese were at risk for severe depression (aOR = 0.40; p = 0.033). The BMI was associated with weight misperception: adolescents with normal weight tend to overestimate their weight (obesity aOR  =  0.13, p  =  0.017; overweight aOR  =  0.1, p  =  0.001).

**Conclusion:**

There was a clear discrepancy between the actual weight status of the teens and their self-perceived weight. Adolescents are still growing both physically and mentally, and forming their self-image. Thus, health promotion practices designed to create accurate perceptions of current body weight need to be part of prevention efforts.

## Introduction

Adolescence is a period characterized changes leading to a greater concern for physical appearance [1]. Misperceptions of one’s weight are common in adolescence and those who misperceive their weight statute tend to engage in unhealthy dieting practices and behaviors that are conducive to obesity [2]. Underestimation of body weight is associated with health issues including depression and certain psychological conditions, that might lead to obesity [3]. The connection between obesity and impaired mental health has been shown to be indirect, and related factors addressed are a lack of physical activity, low self-esteem, weight-based teasing, disordered eating, distorted body weight perception (BWP), and body dissatisfaction [4]. Self-perceived body weight or body weight perception how in which one perceives its body, regardless of its actual size or BMI [5].

Adolescents perceiving themselves as over- or underweight are at a greater risk of depressive symptoms and may experience impaired health outcomes [6]. Recent Tunisian study among adolescents reports rates of overweight of 19.2%, and of obesity of 4.4% [7]. To our knowledge, no studies were made on the BWP among Tunisian adolescents in the last ten years. This study aimed to describe weight status perception and its association with socio-demographic characteristics (SDC) and mental health disorders among adolescents in a low and middle-income country (LMIC).

## Methods

### 1 Study design

The study was conducted as a part of non-communicable diseases (NCDs) risk factors and mental health disorders among adolescents: a cross-sectional study in high schools in the urban area of the governorate of Sousse, Tunisia from January to May 2018 to describe weight status perception and its association with socio-demographic characteristics (SDC) and mental health disorders among adolescents.

### 2 Study population

To guarantee the full representation of all the appropriate age groups and various strata, we selected our sample from all the educational grades within public secondary schools. We included all students, studying in selected classes in selected public high schools and who consent to participate. We did not include adolescents living in institutions for people with physical or intellectual disabilities; who dropped out of school; who left school temporarily or for a long period. Using Epi info version 6 software, the prevalence of weight misperception of 26% [8] was used since it gives us the largest sample size. The precision level was fixed at 4.5% and the cluster effect was 2 since we randomly selected schools and then classes. The estimated sample size was 1095 participants. To assume a 20% for the possible nonresponse the needed sample size was 1314 adolescents.

Study participants were enrolled through a two-stage with cluster selection strategy proportional sampling to get a representative sample:

In the first step, three geographic areas in Sousse were chosen which are Sousse-center, Sousse-Jawhara, and Sousse-Riyadh. From 12 schools, a total of 10 schools were eligible within that area (number of students more than 500). To have the needed sample size, four out of a total of ten eligible secondary schools were randomly selected. In the second step, classes in the selected high schools were stratified by grades (1st (14–15 years old), 2nd (15–16 years old), 3rd (16–17 years old), or 4th year (>17 years old), if the student did not repeat the grade). In order to meet the calculated sample size requirement and guarantee representativeness, bearing in mind that the average number of pupils in each class was 25, 59 classes were randomly selected in all schools to have the needed sample. Adolescents studying in those selected classes were included in the study. Table 1 shows the distribution of our sample in different high schools.

**Table 1:** Distribution of the expected sample size.

Secondary schools	Level	Number of classes	Number of students	Selected Classes	Percentage of participants
**Sousse center Ahmed Noureddine**	1st year	11	270	4	31.7%
	2nd year	11	246	4	
	3rd year	10	233	5	
	4th year	15	344	6	
	**Total**	**47**	**1093**	**19**	**419**
**Sousse Jawhara Pioneer**	1st year	8	227	3	26.6%
	2nd year	9	246	3	
	3rd year	10	230	4	
	4th year	11	215	5	
	**Total**	**38**	**915**	**15**	**343**
**Sousse Jawhara Abdelaziz El Bahi**	1st year	9	236	4	21%
	2nd year	7	176	2	
	3rd year	6	126	3	
	4th year	8	183	4	
	**Total**	**30**	**721**	**13**	**278**
**Riyadh Ibn Roched**	1st year	7	175	3	20.7%
	2nd year	5	158	2	
	3rd year	7	147	3	
	4th year	10	232	4	
	**Total**	**29**	**712**	**12**	**274**
**Total**		**144**	**3444**	**59**	**1314**

### 3 Data collection and procedure

A pre-tested questionnaire in Arabic was self-administered in classes by previously trained investigators. Before data collection, the team explained the study’s purposes while simultaneously dispelling any doubts students might have regarding their data confidentiality and anonymity.

We collected the following information:

- **Socio-demographic characteristics**: age, gender, level of education, parents ’level of education, and parents’ professional status.
- **The perception of weight.**
- **Mental health:** The scales enclosed in the questionnaire are designed to evaluate certain mental disorders notably: self-esteem, depression, anxiety, and alexithymia. Each scale was preceded by a brief clarification to facilitate the form-filling.

The different scales used in our study are:

➢ **Self-esteem**: According to Rosenberg’s Self-esteem Scale (RSE) [9].
➢ **Depression**: according to the Beck Depression Inventory-II scale (BDI-II scale) [10].
➢ **Alexithymia**: According to the Twenty-item Toronto Alexithymia Scale [11].
➢ **Anxiety**: According to the Screen for Child Anxiety-Related Disorders (SCARED-C) [8].

A physical examination was performed to collect the following data:

- ➢ **Body weight**: recorded using a portable digital scale previously calibrated with accuracy close to 100 grams. Students were stripped of their shoes and wearing light clothes. The participant stands on the scale symmetrically and remains motionless until the measurement stabilizes. We used a portable electronic scale (Beurer GmbH Soflinger Str.218,89077Ulm, Germany, Type PS160, Max: 180Kg, d = 100g)
- ➢ **The height**: we used a portable wooden stadiometer. The standing height was measured with an accuracy of 0.5 cm on these very barefoot subjects, feet seemed flat together on the floor, back, buttocks, and heels were applied against the wall and the head was placed in a horizontal position so that the line of vision is perpendicular to the body. The moving part of the board is brought back into contact with the head, excluding the hair.

### 4 Variable definitions

- **Adolescent**: a range of age of 10–24 years was used to define an adolescent [1].
- **Body mass index (BMI):** BMI was calculated as weight (kg) divided by the height (m^2^) and categorized according to Cole criteria according to age and gender [12] to determine weight status
- **Obesity and overweight**: WHO defines excess weight as “an abnormal or excessive accumulation of fat which can be harmful to health”. Based on the criteria of the International Obesity Task Force (IOTF), the body mass index (BMI) thresholds defining overweight and obesity were those proposed by Cole et al [12]. An adolescent is considered overweight or obese if their BMI is greater than or equal to the threshold values for age and sex [12].
- **Weight perception according to body image and perception categories:** Is a self-reported category, assessed through visual figures of children with different body constitutions. Participants were asked to tick on the image that best corresponded to their body size [13]:
  - **Perceived body image (Weight perception according to body image)**
    ➢ **Underweight**: if the participant believes he looks like picture 1 or 2 and ticks on one of them.
    ➢ **Normal weight**: if the participant thinks he corresponds to either photo number 3 or 4
    ➢ **Overweight**: if the student picks photo number 5 or 6
    ➢ **Obese:** When the student chooses the last photo (number 7).
  - **Perception categories:** We used their calculated BMI to qualify objectively their perception of their body weight, and whether teens were able to recognize their weight status or not.
    ➢ **Over-estimation**: if the participant ticks on a body diagram corresponding to a weight greater than that measured inside the classrooms.
    ➢ **Good or normal estimation**: if the participant chooses a body diagram corresponding to a weight almost equal to the value of the measured
    ➢ **Under-estimation**: if the participant reports a weight lower than the measured
- **Regular physical activity**: In our study, we considered students doing regular physical activity, all those who exercise at least 60 minutes per day, from moderate to vigorous physical activity for at least 5 days per week.
- **Anxiety disorder:** is assessed using an Arabic version of the Screen for Child Anxiety-Related Disorders (SCARED-C) scale [8]. This scale includes a total of 41 questions treating five subscales (panic disorder, generalized anxiety, separation anxiety, social phobia, and school avoidance): participants answer as follows: 0 (rarely), 1 (sometimes), and 2 (often). The total score may range from 0 to 82 [8]. An overall score ≥25 may presume the existence of an anxiety disorder.
- Panic disorder may be indicated for a score ≥ 7 for items 1, 6, 9, 12, 15, 18, 19, 22, 24, 27, 30, 34, 38.
- Generalized anxiety disorder may be suspected for a score ≥ 9 for items 5, 7,14, 21, 23, 28, 33, 35, 37.
- separation anxiety may be suspected for a score ≥ 5 for items 4, 8, 13, 16, 20, 25, 29, 31.
- social phobia for a score ≥8 for items 3, 10, 26, 32, 39, 40, 41.
- school avoidance may be suggested for a score ≥3 for items 2, 11, 17, and 36 points.

Cronbach’s alfa coefficient in our study was 0.91.

- **Depression**: was measured by the Arabic version of the “Beck Depression Inventory-II” scale (BDI-II scale) which assesses depression during the previous week with 42 items rated on a 4-point Likert scale [10]. The total score can range from 0 to 39 [10]. Nno depression was posed for a score of 0 to 3; mild depression for 4 to 7; moderate depression for 8 to 15 and severe depression for a score ≥ 16 [10]. The Cronbach’s alfa coefficient in our study was 0.84.
- **Alexithymia:** is defined as the inability to identify and describe one’s feelings [14]. It was measured by the Arabic-validated version of "The twenty-item Toronto Alexithymia Scale". The scale includes 21 items rated from 1 to 5 according to the Likert scale. A score between 51–60 indicates possible alexithymia and a score ≥ 61 means alexithymia. Cronbach’s alfa coefficient in our study was 0.8.
- **Self-esteem**: The Rosenberg Self-esteem Scale (RSE) measures self-esteem with 5 positive items (1, 3, 4, 7, and 10) and 5 negative items (2, 5, 6, 8, and 9) [9], engendering a final score of 10 to 40. The lower the positive results, the higher the self-esteem. Cronbach’s alfa coefficient in our study was 0.7.

### 5 Statistical analyses

Statistical analyses were performed using the SPSS statistical package (version 20.0, SPSS Inc, Chicago, IL, USA). Quantitative variables were presented with means and standard deviation. Qualitative variables were presented by percentages and numbers. The chi-square test was used to compare qualitative variables and one-way Anova test was used to compare means. The Significance level was set as a p-value < 0.05.

Multinomial logistic regression was used to assess the associated factors to weight perception categories with 1 = underweight (reference category), 2 = normal weight, 3 = overweight, and 3 = obesity. Variables with a significance level of p< 0.2 were included in the model.

### 6 Ethics approval and consent to participants

The study protocol was approved by the Farhat Hached University Hospital ethics committee, Sousse, Tunisia (IRB00008931). The study was done by the declaration of Helsinki and under the highest ethical standards. Written informed consent was obtained from all subjects and their legal guardian(s) before data collection. Also, before height and weight examination, participants’ oral informed consent was obtained.

## Results

### 1 Socio-demographic characteristics

Four secondary schools were included (one in Sousse Ville, two in Sousse Jawhara and one in Sousse Riadh). The total number of students participating was 1399 with a response rate of 86.68% (Table 1). The female sex was predominant with a prevalence of 60.5% and a Male /Female sex ratio of 0.65. The mean age was 17.03 ± 1.51 year. The predominant age group was between 14 and 16 years (38.2%). (Table 2). The four levels of study were similarly represented in our study. Of the participants, 29.1% repeated at least one year during their studies (Table 2).

**Table 2:** Socio-demographic characteristics (SDC) of adolescents and weight categories and perception among our study population.

VARIABLES		N(%)
**Socio-demographic characteristics**		
**Gender (n = 1399)**	Male	553(39.5)
	Female	846(60.5)
**Classes (n = 1399)**	First-year	368(26.3)
	Second year	304(21.7)
	Third year	354(25.3)
	Fourth-year	373(26.7)
**Section (n = 1399)**	Commune section	366(26.2)
	Sciences	312(22.3)
	Letter	193(13.8)
	Computing	87(6.2)
	Mathematics	119(8.5)
	Economy	209(14.9)
	Technical	113(8.1)
**Repeating grade (n = 1399)**	Yes	407(29.1)
	No	992(70.9)
**Mother’s level of education (n = 1381)**	Illiterate or primary	388(28.1)
	Secondary	489(35.4)
	University or master’s degree	504(36.5)
**Father’s level of education (n = 1381)**	Illiterate or primary	326(23.6)
	Secondary	480(34.8)
	University or master’s degree	575(41.6)
**Mother’s occupation (n = 1363)**	Housewife	683(50.1)
	Factory girl	121(8.9)
	liberal profession	106(7.8)
	State officer	328(24.1)
	Senior	125(9.2)
**Father’s occupation (n = 1297)**	No occupation	81(6.2)
	Worker	211(16.3)
	liberal profession	377(29.1)
	State officer	399(30.8)
	Senior	299(17.7)
**Weight categories and weight perception among our study population**		
**Measured Weight categories**	Normal weight	1016(72.6)
	Overweight	286(20.4)
	Obese	97(7.0)
**Weight perception according to body image**	Underweight	478(34.5)
	Normal weight	568(41.0)
	Overweight	230(16.6)
	Obese	110(7.9)
**Perception category**	Under-estimation	632(45.6)
	Good estimation	625(45.1)
	OVER-ESTIMATION	129(9.3)

### 2 Measured weight status and weight perception

Regarding the measured weight category, the majority of our sample was classified as normal weight (71.8%) (Table 2). The mean height was 167.77± 8.74 cm. Among adolescents, 34.5% perceived themselves as underweight, 41% as normal weight, 16.6% as overweight, and 7.9% as obese. When compared to the measured weight category, 45.6% of participants underestimated their weight, 9.3% overestimated, and 45.1% correctly estimated their weight.

### 3 Association between SDC and perceived weight categories according to body image

Overall, no significant difference was found between weight perception categories and gender or age (p = 0.089 and p = 0.123 respectively), according to the univariate analysis. Among adolescents whose have a primary level of education, 40.1% perceived themselves as underweight and 41.7% as normal weight. Regarding participants having a mother with a high school level of education, 38.2% perceived themselves as underweight, 38.4% normal weight, 15.3% overweight and 6.6% obese. Among adolescents whose mothers had university education, 26.7% considered themselves as underweight and 6.6% obese. Results show a significant association between mother’s level of education and child’s weight perception (p<10^−3^). A significant association was also found between adolescents’ weight perception, father’s level of education (p = 0.005), and mother’s occupation (p = 0.022) (Table 3).

**Table 3:** Factors associated with weight perception: Univariate analysis.

Variables		Perceived weight				p-value
		Under Weight	Normal weight	Over weight	obese	
**Gender n (%)**	Male	177(32.4)	246(45.0)	87(15.9)	37(6.8)	0.089
	Female*	301(35.9)	322(38.4)	143(17.0)	73(8.7)	
	OR(95%CI)	*	1.30(1.01–1.67	1.04(0.75–1.43)	0.86(0.56–1.33)	
**Age category n (%)**	[14–16]*	173(32.6)	230(43.3)	91(17.1)	37(7.0)	0.431
	[16–17]	121(35.5)	139(40.8)	58(170)	23(6.7)	
	OR(95%CI)	*	0.86(0.63–1.18)	0.91(0.61–1.36)	0.89(0.50–1.57)	
	>17	184(35.8)	199(38.7)	81(15.8)	50(9.7)	
	OR(95%CI)	*	0.81(0.61–1.08)	0.84(0.58–1.21)	1.27(0.79–2.04)	
**Mean age m(sd)**		17.1(1.53)	16.9(1.54)	16.97(1.42)	17.24(1.45)	0.123
**Grade n (%)**	1st year	107(29.4)	166(45.6)	62(17.0)	29(8.0)	0.101
	2nd year	117(38.6)	121(39.9)	47(15.5)	18(5.9)	
	OR(95%CI)	*	0.67(0.47–0.95)	0.70(0.44–1.10)	0.57(0.30–1.08)	
	3rd year	127(36.5)	134(38.5)	64(18.4)	23(6.6)	
	OR(95%CI)	*	0.68(0.48–0.96)	0.87(0.56–1.34)	0.67(0.37–1.22)	
	4th year	127(34.2)	147(39.6)	57(15.4)	40(10.8)	
	OR(95%CI)	*	0.75(0.53–0.96)	0.78(0.50–1.21)	1.16(0.68–2.00)	
**Repeating Grades n (%)**	Yes*	143(35.7)	157(39.2)	60(15.0)	41(10.2)	0.150
	No	335(34.0)	411(41.7)	170(17.3)	69(7.0)	
	OR(95%CI)	-	1.12(0.86–1.46)	1.21(0.85–1.72)	0.72(0.47–1.11)	
**Mother’s Level of education n(%)**	Illiterate/Primary*	154(40.1)	160(41.7)	40(10.4)	30(7.8)	<0.001
	OR(95%CI)					
	Highschool	187(38.2)	172(35.2)	85(17.4)	45(9.2)	
	OR(95%CI)	-	0.89(0.65–1.20)	1.80(1.14–2.69)	1.24(0.74–2.05)	
	University	134(26.7)	233(46.4)	102(20.3)	33(6.6)	
	OR(95%CI)	-	1.67(1.23–2.27)	2.9(1.90–4.52	1.26(0.73–2.18	
**Father’s level of education n (%)**	Illiterate/Primary	118(36.2)	125(38.3)	47(14.4)	36(11.0)	0.005
	OR(95%CI)					
	Highschool	183(38.4)	183(38.4)	73(15.3)	38(8.0)	
	OR(95%CI)		0.94(0.68–1.06)	1.00(0.65–1.55)	0.67(0.39–1.12)	
	University	172(30.1)	258(45.2)	106(18.6)	35(6.1)	
	OR(95%CI)		1.42(1.03–1.94	1.58(1.02–2.35)	0.68(0.41–1.14)	
**Mother’s occupation n (%)**	Housewife*	265(38.0)	270(38.7)	107(15.4)	55(7.9)	0.089
	factory girl	45(37.8)	45(37.8)	18(15.1)	11(9.2)	
	OR(95%CI)	*	0.98(0.63–1.53)	0.99(0.55–1.79)	1.19(0.74–1.94)	
	Private practice	31(29.2	46(43.4)	19(17.9)	10(9.4)	
	OR(95%CI)	*	1.46(0.90–2.37)	1.52(0.82–2.80)	1.55(0.72–3.36)	
	State officer or an executive	129(28.6)	204(45.2)	86(19.1)	32(7.1)	
	OR(95%CI)	*	1.55(1.18–2.06)	1.65(1.16–2.35)	1.18(0.57–2.42)	
**Father’s occupation n (%)**	Doesn’t* work	23(29.1)	34(43.0)	14(17.7)	8(10.1)	0.555
	Laborer	72(34.3)	91(43.3)	29(13.8)	18(8.6)	
	OR(95%CI)	*	0.86(0.46–1.58)	0.66(0.30–1.47)	0.72(0.28–1.87)	
	Private practice	137(36.6)	139(37.2)	72(19.3)	26(7.0)	
	OR(95%CI)	*	0.69(0.39–1.23)	0.86(0.42–1.78)	0.55(0.22–1.35)	
	State officer or an executive	207(33.1)	271(43.4)	100(16.0)	46(7.4)	
	OR(95%CI)	*	0.89(0.51–1.55)	0.79(0.39–1.61)	0.64(0.27–1.52)	
**DEPRESSION**	Absent/minimal*	162(33.9)	219(38.6)	74(32.2)	29(26.4)	<0.001
	Mild	95(19.9)	136(23.9)	51(22.2)	21(19.1)	
	OR(95%CI)		1.06(0.76–1.48)	1.18(0.76–1.82)	1.24(0.67–2.29)	
	Moderate	159(33.3)	179(31.5)	75(32.6)	37(33.6)	
	OR(95%CI)	*	0.83(0.62–1.12)	1.03(0.70–1.52)	1.30(0.76–2.21)	
	Severe	62(13.0)	34(6.0)	30(13.0)	23(20.9)	
	OR(95%CI)	*	0.41(0.26–0.65)	1.06(0.63–1.77)	2.07(1.11–3.86)	
**SELF-ESTEEM**	Low*	180(37.7)	168(29.6)	99(43.0)	55(50.0)	<0.001
	Average	198(41.4)	257(45.2)	82(35.7)	39(35.5)	
	OR(95%CI)	*	1.40(1.05–1.84)	0.75(0.53–1.07)	0.65(0.41–1.02)	
	High	100(29.9)	143(25.2)	49(21.3)	16(14.5)	
	OR(95%CI)	*	1.53(1.10–2.13)	0.75(0.53–1.07)	0.65(0.41–1.02)	
**ANXIETY**	Yes*	330(69.0)	360(63.4)	162(70.4)	89(80.9)	0.002
	No	148(31.0)	208(36.6)	68(29.6)	21(19.1)	
	OR(95%CI)	*	1.29(0.99–1.67)	0.94(0.66–1.32)	0.53(0.32–0.88)	
Panic disorder	Yes*	289(60.5)	316(55.6)	142(61.7)	75(68.2)	0.055
	No	189(39.5)	252(44.4)	88(38.3)	35(31.8)	
	OR(95%CI)	*	1.22(0.95–1.56)	0.95(0.69–1.01)	0.71(0.46–1.11)	
Generalized anxiety disorder	Yes*	222(46.4)	249(43.8)	117(50.9)	68(61.8)	0.004
	No	256(53.6)	319(56.2)	113(49.1)	42(38.2)	
	OR(95%CI)	*	1.11(0.87–1.42)	0.84(0.61–1.15)	0.54(0.35–0.82)	
Separation anxiety	Yes*	277(57.9)	300(52.8)	132(57.4)	73(66.4)	0.047
	No	201(42.1)	268(47.2)	98(42.6)	37(33.6)	
	OR(95%CI)	*	1.233(0.96–1.57)	1.02(0.74–1.41)	0.70(0.45–1.08)	
Social anxiety disorder	Yes*	176(36.8)	195(34.3)	92(40.0)	46(41.8)	0.297
	No	302(63.2)	373(65.7)	138(60.0)	64(58.2)	
	OR(95%CI)	*	1.12(0.86–1.44)	0.88(0.63–1.21)	0.81(0.53–1.24)	
Significant school avoidance score	Yes*	195(40.8)	198(34.9)	89(38.7)	110(48.2)	0.035
	No	283(59.2)	198(65.1)	141(61.3)	57(51.8)	
	OR(95%CI)	*	1.29(1.00–1.66)	1.09(0.79–1.51)	0.74(0.49–1.12)	
**ALEXITHYMIA**	Yes	337(70.5)	398(70.1)	178(77.4)	85(77.3)	0.093
	No	141(29.5)	170(29.9)	52(22.6)	25(22.7)	
	OR(95%CI)		1.02(0.78–1.33)	0.70(0.48–1.01)	0.70(0.43–1.14)	

M (sd): mean and standard deviation

* reference category

### 4 Association between SDC, perceived weight categories and mental health

Adolescents perceiving themselves as obese were at risk for severe depression in 20.9% of cases, compared to 13% in overweight perception group, 6% in normal weight and 13% in underweight perception group. In our study, the association between depression and weight perception was significant with p value < 10^−3^ (Table 3).

Among obese weight perception group, 50% of participants reported low self-esteem, compared to 43% in overweight perception group, 29.6% in normal weight perception and 37.7% in underweight perception group (p< 10^−3^). Anxiety disorder was also reported in 80.9% of cases among adolescents perceiving themselves as obese compared to 70.4% in adolescents perceiving themselves as overweight, 63.4% among normal weight and 69% among underweight perception group (p = 0.002). Our study did not show a significant association between alexithymia and weight perception in adolescents (p = 0.093) (Table 3).

### 5 Multivariate analysis

Perceived weight categories were associated with socio-demographic factors such as gender and maternal educational level, as shown in Table 4. The BMI was associated with weight misperception: adolescents with normal weight tend to overestimate their weight (obesity aOR  =  0.14, p  =  0.016; overweight aOR  =  0.1, p  =  0.001). Findings related to mental health showed that only depression was significantly associated with weight misperception. Adolescents perceiving themselves as obese were at risk for severe depression (aOR = 0.40; p = 0.033).

**Table 4:** Results of multinomial logistic regression among adolescents in Sousse Tunisia, 2017–2018.

Variables	Weight perception n (%)								
	Normal weight			Overweight			Obese		
	aOR	CI_95%_	P	aOR	CI_95%_	P	aOR	CI_95%_	P
**Sex (male)**	1.37	1.05–1.79	**0.021**	1.52	1.02–2.25	**0.042**	0.94	0.53–1.69	0.842
**Mother’s Education**									
University	1,56	1.13–2.15	**0.006**	2.50	1.51–4.12	**<0.001**	0.92	0.46–1.84	0.822
Highschool	0.84	0.61–1,15	0.277	1.50	0.91–2.48	0.115	0.93	0.48–1.79	0.823
Illiterate/Primary *	-	-	-	-	-	-	-	-	-
**Depression **									
Severe	0.86	0.30–0.79	**0.004**	1.51	0.79–2.88	0.218	2.50	1.08–5.79	**0.033**
Moderate	0.98	0.71–1.34	0.891	1.30	0.81–2.09	0.282	1.60	0.81–3.18	0.175
Mild	1.29	0.91–1.83	0.146	1.83	1.09–3.09	**0.022**	1.90	0.88–4.07	0.100
Absent/minimal *	-	-	-	-	-	-	-	-	-
**Measured weight category**									
Obese	4.07	0.86–19.39	0.077	10.76	5.42–20.14	**<0.001**	33.91	17.24–42.0	**<0.001**
Overweight	16.22	7.80–33.75	**<0.001**	20.77	15.36–30.67	**<0.001**	25.15	17.61–35.52	**<0.001**
Normal weight *	-	-	-	-	-	-	-	-	-

a: adjusted odds ration

CI: confident interval

*Reference category

## Discussion

While many previous studies focused on investigating associated factors between weight status and mental health disorders [4, 15], focus of our study was primarily on weight status perception and its connection with mental health disorders and socio-demographic characteristics among adolescents in a low and middle-income country (LMIC).

Obese weight misperceivers were less likely to be trying to lose weight or exercise, and more likely to be trying to stay the same or doing nothing [16, 17], which identifies accurate weight perception as an important step in changing weight related behaviors [17, 18]. Furthermore, the accurate assessment of weight status was identified as a precondition to the effectiveness of interventions for overweight and obese individuals [17]. Finally, weight underestimation was reported to be more common in families and communities with lower socioeconomic conditions [6], and given the fact our study was conducted in LMIC, the latter result may be considered consistent with our findings. According to Minsky et al., one of the reasons for weight underestimation may also be that the social milieu has become increasingly overweight, i.e. around 60% of US general population is overweight or obese [19]. It is important to highlight that in order to curb exponential rise of child obesity, it may be crucial to correct weight underestimation first.

According to our results, body weight perception was shown to be significantly associated with potential mental health issues: depression, anxiety, and low self-esteem. In the same way, those who perceived themselves as obese, scored lowest on self-esteem scales, and were shown to be at much higher risk for anxiety. These statements are consistent with those of Ramos et al. [20] where overweight body image perception and low body image satisfaction were connected with anxious/depressive, and withdrawn/depressive symptoms. Şanlier et al. [21] also found a negative relationship between body image scores and depression scores. Some studies have shown that there are no connections between actual weight and mental health disorders; instead, mental health disorders were shown to be connected with the perceived weight [22]. People who misperceive their weight are more likely to suffer from mental illnesses, but also those who already suffer from mental illness are more likely to misperceive their weight, compared to the general population [19]. Bodyweight perception was shown to be a more substantial predictor of adolescent distress and a wide range of psychological problems than the BMI. In fact, subjective happiness was lower, and subjective stress, sadness/despair, suicidal ideation, and suicide planning were higher in adolescents overestimated their weight than those who have a normal estimation of their body weight [23].

Weight overestimation and dissatisfaction with one’s weight was previously shown to be connected with depressive symptoms, anxiety, and reduced self-esteem [24], as well as with compensatory weight-loss behaviors [18, 25]. Moreover, perceived overweight was shown to be implicated in the development of obesity [26–28]. In fact, weight misperception may lead to changing in either eating habits or physical activity [26]. Extreme weight control behaviors, such as use of diet pills, fasting, skipping meals, use of food supplements were all shown to be associated with increased weight gain in children and adolescents [28–30].

### Strengths and limitations

Because of the cross-sectional design of the study, no causal relations between weight perception and mental health disorders can be established. However, to our knowledge, no other study with an important representative sample size was performed in our context to access weight perception and associated factors, especially mental health disorders, among adolescents. So, based on the assumptions made by this study, further longitudinal studies are needed to determine causal links between mental health disorders and body weight perception.

The majority of collected data were self-reported by participants which may overestimate or underestimate some variables. We did not collect data related to sexual maturation. However, to ensure the validity of our results, we used validated scales and pretested questionnaires. Body weight and height were also measured by trained health professionals using standardized tools.

## Conclusion

This study found that there was a clear discrepancy between the actual weight status of the teens and their self-perceived weight. Significant association was found between weight perception and depression according to our multinomial regression. Underestimation may negatively affect the efficacy of obesity prevention efforts because often the decision to lose weight is based as much on one’s self-perception of being.

## Supporting information

S1 File (SAV)

## References

[1] Sawyer SM, Azzopardi PS, Wickremarathne D, Patton GC. The age of adolescence…and young adulthood–Authors’ reply. Lancet Child Adolesc Health. avr 2018;2(4):e7. 10.1016/S2352-4642(18)30075-0 30169305

[2] Graber JA, Sontag LM. Internalizing problems during adolescence. In: Handbook of adolescent psychology: Individual bases of adolescent development, Vol 1, 3rd ed. Hoboken, NJ, US: John Wiley & Sons Inc; 2009. p. 642–82.

[3] Kokka I, Mourikis I, Bacopoulou F. Psychiatric Disorders and Obesity in Childhood and Adolescence—A Systematic Review of Cross-Sectional Studies. Children. 1 févr 2023;10(2):285. doi: 10.3390/children10020285 36832413 PMC9955505

[4] Hunger JM, Major B. Weight stigma mediates the association between BMI and self-reported health. Health Psychol. 2015;34(2):172–5. doi: 10.1037/hea0000106 25133837 PMC4308542

[5] Park B, Cho HN, Choi E, Seo DH, Kim S, Park YR, et al. Self-perceptions of body weight status according to age-groups among Korean women: A nationwide population-based survey. Kim Y, éditeur. PLOS ONE. 17 janv 2019;14(1):e0210486. 10.1371/journal.pone.0210486 PMC6336301 30653596

[6] Park E. Overestimation and Underestimation: Adolescents’ Weight Perception in Comparison to BMI-Based Weight Status and How It Varies Across Socio-Demographic Factors. J Sch Health. févr 2011;81(2):57–64. doi: 10.1111/j.1746-1561.2010.00561.x 21223272

[7] Regaieg S, Charfi N, Elleuch M, Mnif F, Marrakchi R, Yaich S, et al. Obésité, activité physique et temps de sédentarité chez des adolescents scolarisés, âgés de 15 à 18 ans de la ville de Sfax (Tunisie). Pan Afr Med J [Internet]. 2015 [cité 7 avr 2023];22. Disponible sur: http://www.panafrican-med-journal.com/content/article/22/370/full/10.11604/pamj.2015.22.370.6121 PMC4789185 27022430

[8] Birmaher B, Khetarpal S, Brent D, Cully M, Balach L, Kaufman J, et al. The Screen for Child Anxiety Related Emotional Disorders (SCARED): Scale Construction and Psychometric Characteristics. J Am Acad Child Adolesc Psychiatry. avr 1997;36(4):545–53. 9100430 10.1097/00004583-199704000-00018

[9] Vasile C. Is the Body Image So Important? Physical Identity in Relation to Gender and Self Esteem. Procedia—Soc Behav Sci. août 2015;203:443–7.

[10] West J. An Arabic Validation of a Depression Inventory. Int J Soc Psychiatry. déc 1985;31(4):282–9. doi: 10.1177/002076408503100406 4077428

[11] El Abiddine FZ, Dave H, Aldhafri S, El-Astal S, Hemaid F, Parker JDA. Cross-validation of the 20-item Toronto Alexithymia Scale: Results from an Arabic multicenter study. Personal Individ Differ. juill 2017;113:219–22.

[12] Cole TJ. Establishing a standard definition for child overweight and obesity worldwide: international survey. BMJ. 6 mai 2000;320(7244):1240–1240. doi: 10.1136/bmj.320.7244.1240 10797032 PMC27365

[13] Hsu Ya-Wen, Liou Tsan-Hon, YiIng Mei Liou Hsin-Jen Chen, Chien Li-Yin. Measurements and profiles of body weight misperceptions among Taiwanese teenagers: a national survey. Asia Pac J Clin Nutr. 1 janv 2016;25(1). doi: 10.6133/apjcn.2016.25.2.08 26965769

[14] Parker JDA, Taylor GJ, Bagby RM. The 20-Item Toronto Alexithymia Scale: III. Reliability and factorial validity in a community population. J Psychosom Res. 1 sept 2003;55(3):269–75. 12932802 10.1016/s0022-3999(02)00578-0

[15] Halfon N, Larson K, Slusser W. Associations Between Obesity and Comorbid Mental Health, Developmental, and Physical Health Conditions in a Nationally Representative Sample of US Children Aged 10 to 17. Acad Pediatr. janv 2013;13(1):6–13. doi: 10.1016/j.acap.2012.10.007 23200634

[16] Mbogori T, Arthur TM. Perception of Body Weight Status Is Associated With the Health and Food Intake Behaviors of Adolescents in the United States. Am J Lifestyle Med. mai 2021;15(3):347–55. doi: 10.1177/1559827619834507 34025327 PMC8120624

[17] Rutten L, Bodde A, Beebe TJ, Chen LP, Perez-Vergara K, Ziegenfuss JY, et al. Misperceptions of weight status among adolescents: sociodemographic and behavioral correlates. Patient Relat Outcome Meas. déc 2014;163. doi: 10.2147/PROM.S72621 25525400 PMC4266328

[18] Haynes A, Kersbergen I, Sutin A, Daly M, Robinson E. A systematic review of the relationship between weight status perceptions and weight loss attempts, strategies, behaviours and outcomes: Perceived overweight and weight management. Obes Rev. mars 2018;19(3):347–63. 10.1111/obr.12634 PMC5814847 29266851

[19] Minsky S, Vreeland B, Miller M, Gara M. Concordance Between Measured and Self-Perceived Weight Status of Persons With Serious Mental Illness. Psychiatr Serv. janv 2013;64(1):91–3. doi: 10.1176/appi.ps.201100515 23280463

[20] Ramos P, Moreno-Maldonado C, Moreno C, Rivera F. The Role of Body Image in Internalizing Mental Health Problems in Spanish Adolescents: An Analysis According to Sex, Age, and Socioeconomic Status. Front Psychol. 22 août 2019;10:1952. doi: 10.3389/fpsyg.2019.01952 31507499 PMC6714592

[21] Şanlier N, Yabanci N, Alyakut Ö. An evaluation of eating disorders among a group of Turkish university students. Appetite. nov 2008;51(3):641–5. doi: 10.1016/j.appet.2008.05.058 18584912

[22] Ren L, Xu Y, Guo X, Zhang J, Wang H, Lou X, et al. Body image as risk factor for emotional and behavioral problems among Chinese adolescents. BMC Public Health. déc 2018;18(1):1179. doi: 10.1186/s12889-018-6079-0 30326854 PMC6192148

[23] Lee KH, Bong SH, Kang DH, Choi TY, Kim JW. Association Between Weight Misperception and Some Mental Health-Related Characteristics in Korean Adolescents. Neuropsychiatr Dis Treat. déc 2020;Volume 16:3053–62. doi: 10.2147/NDT.S286470 33328734 PMC7735779

[24] Johnson F, Wardle J. Dietary Restraint, Body Dissatisfaction, and Psychological Distress: A Prospective Analysis. J Abnorm Psychol. févr 2005;114(1):119–25. doi: 10.1037/0021-843X.114.1.119 15709818

[25] Alkazemi D, Zafar TA, Ebrahim M, Kubow S. Distorted weight perception correlates with disordered eating attitudes in Kuwaiti college women. Int J Eat Disord. mai 2018;51(5):449–58. doi: 10.1002/eat.22852 29488236 PMC6586007

[26] San Martini MC, de Assumpção D, Barros MB de A, de A Barros Filho A, Mattei J. Weight self-perception in adolescents: evidence from a population-based study. Public Health Nutr. mai 2021;24(7):1648–56. doi: 10.1017/S1368980021000690 33648614 PMC10195491

[27] Cuypers K, Kvaløy K, Bratberg G, Midthjell K, Holmen J, Holmen TL. Being Normal Weight but Feeling Overweight in Adolescence May Affect Weight Development into Young Adulthood—An 11-Year Followup: The HUNT Study, Norway. J Obes. 2012;2012:1–8. 10.1155/2012/601872 PMC3362140 22666556

[28] Neumark-Sztainer DR, Wall MM, Haines JI, Story MT, Sherwood NE, van den Berg PA. Shared Risk and Protective Factors for Overweight and Disordered Eating in Adolescents. Am J Prev Med. nov 2007;33(5):359–369.e3. doi: 10.1016/j.amepre.2007.07.031 17950400

[29] Lankinen V, Fröjd S, Marttunen M, Kaltiala-Heino R. Perceived rather than actual overweight is associated with mental health problems in adolescence. Nord J Psychiatry. 17 févr 2018;72(2):89–96. doi: 10.1080/08039488.2017.1389987 29124989

[30] Niemeier HM, Raynor HA, Lloyd-Richardson EE, Rogers ML, Wing RR. Fast Food Consumption and Breakfast Skipping: Predictors of Weight Gain from Adolescence to Adulthood in a Nationally Representative Sample. J Adolesc Health. déc 2006;39(6):842–9. doi: 10.1016/j.jadohealth.2006.07.001 17116514

